# Elemental Diet Enriched with Amino Acids Alleviates Mucosal Inflammatory Response and Prevents Colonic Epithelial Barrier Dysfunction in Mice with DSS-Induced Chronic Colitis

**DOI:** 10.1155/2020/9430763

**Published:** 2020-08-14

**Authors:** Di Guo, Jun Yang, Fangmei Ling, Lei Tu, Junrong Li, Yidong Chen, Kaifang Zou, Liangru Zhu, Xiaohua Hou

**Affiliations:** ^1^Division of Gastroenterology, Union Hospital, Tongji Medical College, Huazhong University of Science and Technology, 1277 Jiefang Avenue, Wuhan, 430022 Hubei, China; ^2^Department of Urology, Union Hospital, Tongji Medical College, Huazhong University of Science and Technology, 1277 Jiefang Avenue, Wuhan, 430022 Hubei, China

## Abstract

**Background:**

Clinical data suggest that enteral nutrition (EN) effectively decreases disease activity and maintains remission in patients with inflammatory bowel disease (IBD). However, the modulatory effects of EN on the intestinal mucosal immune system remain unclear.

**Aims:**

This study first aimed at comparing the therapeutic effects of three EN formulas on ameliorating dextran sulfate sodium- (DSS-) induced chronic colitis; with the most effective formula, we then examined its influence on the mucosal inflammatory response and epithelial barrier function.

**Methods:**

The effect of EN formulas on colitis in mice was assessed by body weight, disease activity index scores, colon length, and H&E staining for pathological examination. Colonic and circulating cytokine expression levels and the frequencies of immune cells were also analyzed. Intestinal epithelial barrier function was evaluated by detecting tight junction proteins.

**Results:**

We found that among the three EN formulas, an elemental diet (ED) containing enriched amino acids restored the colitis-related reduction in body weight better than the other two EN formulas. ED amino acids suppressed the release of colonic proinflammatory mediators and maintained the expression of tight junction proteins in these mice. ED amino acid treatment mitigated the colitis-induced increase in CD103^+^CD11b^+^ dendritic cells and CD4^+^ and CD8^+^ T cells and inhibited the predominant Th1/Th17 responses particularly in the colonic mucosal lamina propria of mice with colitis.

**Conclusions:**

We showed that ED amino acids can be an effective immunomodulatory agent to reduce colitis-related inflammation by inhibiting proinflammatory mediators and Th1/Th17 cell responses and by repairing the disrupted epithelial barrier.

## 1. Introduction

Nutritional disturbances represent a frequent manifestation in inflammatory bowel disease (IBD) patients, especially those with Crohn's disease (CD) [1]. There is increasing evidence that dietary factors might play a role in the pathogenesis of IBD, and especially CD [2]. Moreover, enteral nutrition (EN) has successfully been used as a nutritional therapy for patients with CD. EN remains of interest for patients presenting with malnutrition, those who failed with other therapeutics, patients with complications, or selected patients on long-term maintenance therapy with fewer side effects [3–5]. It has also been used as a first-line therapy for pediatric patients with CD [6] and was shown to induce clinical remission and mucosal healing and improve body composition in patients with CD [7–9].

These data suggested that EN effectively decreases disease activity and maintains remission in patients with IBD, but the potential mechanism is multifactorial, generally due to its anti-inflammatory effects and regulation of the intestinal microflora [1]. However, few studies have addressed the regulatory effects of EN on adaptive and innate immune cell profiles in the immunomicroenvironment of colitis.

It has been previously reported that EN reduces inflammation in murine IL-10^−/−^ cell transfer colitis models [10]. However, the anti-inflammatory effect of different EN formulas in a chronic colitis model remains unclear. In the treatment of IBD, three different types of EN formulas are commonly used, including an elemental diet (ED) containing enriched amino acids, a semielemental formula containing short peptides, and a polymeric formula diet containing intact protein. Meanwhile, studies comparing different EN treatments have obtained conflicting results. An earlier study showed a significantly higher remission rate in acute CD patients treated with ED amino acids than in patients receiving a polymeric diet (75% vs. 36%, *p* < 0.03) [11]. But later observation found that elemental and polymeric diets were equally effective [12]. It was speculated that the nitrogen source and fat content might be relevant to the therapeutic efficacy of EN. However, recent comprehensive studies providing open comparisons of the therapeutic efficacy of these different EN formulas are still lacking.

In the present study, we aimed to examine whether these three EN formulas could ameliorate disease activity in mice with chronic colitis induced by dextran sulfate sodium (DSS) and to compare their therapeutic efficacy. Next, with the most effective EN formula, we further investigated its regulatory effects on immune cell and inflammatory cytokine profiles, as well as intestinal epithelial barrier function, to explore its potential immunological mechanism of action.

## 2. Materials and Methods

### 2.1. Mice and Disease Model

All experiments were approved by the Institutional Animal Care and Use Committee of Tongji Medical College, Huazhong University of Science and Technology, Wuhan, China (S2257). Additionally, the study was performed in accordance with institutional guidelines. Male C57BL/6 mice, 10 weeks of age, were obtained from HFK Bioscience (Beijing, China). The mice were housed in a specific pathogen-free facility at the Experimental Animal Center. They were given a standard chow diet and tap water ad libitum. Experimental chronic colitis was induced by an oral administration of water containing 2% (wt/vol) dextran sodium sulfate (DSS, MP Biomedicals, Illkirch, France) for three cycles (5 days with 2% DSS and 4 days of water). The control and colitis groups of mice were allowed free access to food and water or DSS solution. The mice in the three colitis groups received three different exclusive enteral nutrition formulas (*n* = 10 per group) daily beginning on the 24^th^ day, and all the mice were sacrificed on day 42. All exclusive enteral nutrition diets were isocaloric and equivalent concentrations (every mouse received 20 kcal per day). The exclusive enteral nutrition formulas are as follows: Enteral Nutritional Powder (AA), an amino acid-enriched elemental diet (Wanhe Pharmaceutical Co., Ltd., Shenzhen, China); Enteral Nutritional Suspension (SP), a semielemental formula containing short peptides (Nutricia Pharmaceutical Co., Ltd., Wuxi, China); and Enteral Nutritional Powder (TP), a polymeric formula diet containing intact protein (Abbott Lab., Zwolle, Netherlands). A comparison of three different EN formulas are listed in Table 1, and the composition of the different formulas are listed in Table S1. Disease activity index (DAI) scores were assessed by combining the scores of weight loss, stool consistency, and bleeding [13]. At the end of the experiment, all mice were euthanized, and their colons were collected and photographed.

### 2.2. Hematoxylin-Eosin (H&E) Staining

The medial colon tissues were fixed in 10% buffered formalin overnight and embedded in paraffin. The tissue sections (5 *μ*m) were stained with H&E and examined by light microscopy.

### 2.3. Isolation of Cells

Mouse splenic mononuclear cells (MNCs), mesenteric lymph nodes (MLNs), and lamina propria mononuclear cells (LPMCs) were isolated as previously reported [14]. To isolate LPMCs, in brief, the colonic tissues were flushed and cut into small pieces, followed by washing with 1 mM DTT in Hanks' balanced salt solution at 37°C for 20 min. To remove the epithelium, the pieces were incubated in 1.3 mM EDTA Hank's balanced salt solution at 37°C for 30 min. To isolate LPMCs, the remaining colonic tissue pieces were digested with 0.1 mg/mL Collagenase D (Roche Applied Science, Basel, Switzerland) in serum-free Iscove's modified Dulbecco's medium at 37°C for 1 h and filtered through a cell strainer, which was followed by centrifugation. Subsequently, the LPMCs were enriched by 40%/70% Percoll gradient centrifugation, and the cells at the interface were harvested for further experiments.

### 2.4. Reagents and Flow Cytometric Analysis

Mouse MNCs were stained with the following fluorescence-conjugated surface mAbs: anti-CD3-FITC, anti-CD4-APC, anti-CD8-PE-cy7, anti-NK1.1-PE, anti-CD11b-APC-cy7, anti-CD103-BV421, anti-CD11c-BV510, and anti-CD25-APC antibodies (BD PharMingen, USA). The cells were stained according to the standard procedure of BD PharMingen. Fixation and permeabilization were performed using the Transcription Factor Buffer Set (BD PharMingen, USA), and the cells were then stained with the anti-FoxP3-BV421 intracellular antibodies for 40 min at 4°C. To detect Th1 and Th17 cytokines, MNCs suspended in RPMI 1640 medium supplemented with 10% fetal bovine serum were stimulated with a cell stimulation cocktail (eBioscience, USA) containing phorbol-12-myristate-13-acetate (PMA, 50 ng/mL), ionomycin (1 *μ*g/mL), and monensin (2 *μ*g/mL) at 37°C with 5% CO_2_ for 6 h, and followed by intracellular staining with fluorescently labeled anti-IFN-*γ* and anti-IL-17 antibodies (BioLegend, USA) after washing, fixing, and permeabilizing according to the manufacturer's instructions. Isotype IgGs were used as a control. All samples were detected using a BD LSR Fortessa X-20 Flow Cytometry System (BD, USA) and analyzed using the FlowJo software.

### 2.5. Quantitative Real-Time PCR

Total RNA was extracted from mouse colonic tissue samples using TRIzol reagent (Takara, Shiga, Japan) and then transcribed into cDNA using the ReverTra Ace qPCR RT Kit (Toyobo, Osaka, Japan). The levels of target gene mRNA transcripts relative to the control GAPDH were determined by quantitative RT-PCR analyses on a Roche Light Cycler 480 System using SYBR Green Real-Time PCR Master Mix (Toyobo) and specific primers. The data were analyzed using the 2^−*ΔΔ*Ct^ method. The primers used are as follows: IL-1*β*, forward 5′-CTTCAGGCAGGCAGTATCACTC-3′ and reverse 5′-TGCAGTTGTCTAATGGGAACGT-3′; TNF-*α*, forward 5′-CATCTTCTCAAAATTCGAGTGACAA-3′ and reverse 5′-TGGGAGTAGACAAGGTACAACCC-3′; IFN-*γ* forward 5′-CAGGTGTGATTCAATGACGCT-3′ and reverse 5′-AACTCAAGTGGCATAGATGTGGA-3′; IL-17 forward 5′-CCTCAGACTACCTCAACCGTTC-3′ and reverse 5′-CTCTTCAGGACCAGGATCTCTT-3′; TGF*β*, forward 5′-GACCGCAACAACGCCATCTAT-3′ and reverse 5′-GACAGCCACTCAGGCGTATCAG-3′; IL-10, forward 5′-GGACAACATACTGCTAACCGAC-3′ and reverse 5′-CATGGCCTTGTAGACACCTTG-3′; and GAPDH, forward 5′-CGGATTTGGTCGTATTGGG-3′ and reverse 5′-CTCGCTCCTGGAAGATGG-3′.

### 2.6. Immunofluorescence

The paraffin-embedded mouse colon sections (4 *μ*m) were deparaffinized and stained with primary polyclonal antibodies including anti-rabbit claudin-1, anti-rabbit occludin, and anti-rabbit ZO-1 (all 1 : 100 dilution, Invitrogen, USA) overnight at 4°C, which was followed by secondary staining with Alexa Flour 488 donkey anti-rabbit IgG and then examination under a laser confocal microscopy.

### 2.7. Cytokines Analysis

Serum samples were prepared for the quantification of interferon-*γ* (IFN-*γ*), interleukin-2 (IL-2), interleukin-4 (IL-4), interleukin-6 (IL-6), interleukin-10 (IL-10), interleukin-17A (IL-17A), and tumor necrosis factor-*α* (TNF-*α*) cytokines. Serum samples were analyzed by flow cytometry using the Cytometric Bead Array (CBA) mouse Th1/Th2/Th17 cytokine kit (BD Biosciences, San Jose, USA) according to the manufacturer's instructions. Data were formatted and further analyzed using the BD CBA software.

### 2.8. Statistical Analysis

Statistical analyses were performed using the SPSS version 17.0 and GraphPad Prism version 5.0. The differences between the two groups were analyzed by performing unpaired Student's *t*-tests. Differences among multiple groups were analyzed by one-way analysis of variance (ANOVA). Data are expressed as the means ± SEM. *p* < 0.05 was considered statistically significant.

## 3. Results

### 3.1. An Elemental Diet Enriched in Amino Acids Ameliorates DSS-Induced Chronic Colitis

**Figure 1:**
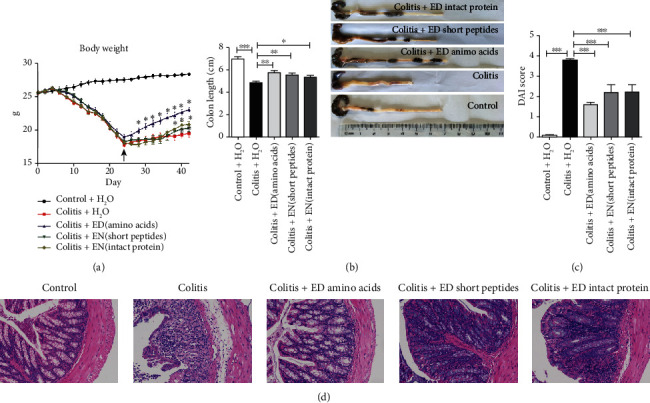
An elemental diet enriched in amino acids ameliorates dextran sulfate sodium- (DSS-) induced chronic colitis. (a) The body weights (g) of individual groups of mice (*n* = 10 per group). (b) Colon length (cm). (c) Disease activity index (DAI) scores. (d) Pathological changes in the colons (H&E staining, magnification, ×200). Data are representative images or expressed as the mean ± SEM of each group from three separate experiments, ∗*p* < 0.05, ∗∗*p* < 0.01, ∗∗∗*p* < 0.001.

After we established a chronic colitis mice model through three cycles of 2% DSS induction, EN amino acids, short peptides, and intact proteins were applied to study their therapeutic effects. Mice with colitis developed severe diarrhea, body weight loss (Figure 1(a)), and bloody stools. A significant reduction in colon length (Figure 1(b)) and an increase in DAI scores (Figure 1(c)) were observed in mice with colitis when compared to those in the control group. H&E staining showed architectural derangements, epithelial necrosis, and diffuse lymphocytic infiltration in the colon of colitis mice (Figure 1(d)). However, the three different ENs resulted in the obvious suppression of colitis severity, including significant reversals in body weight loss and colon length reduction, reduced DAI scores, and decreased lymphocytic infiltration in the colon (Figures 1(a)–1(d)). Between the three EN formulas used to treat colitis mice, although no significant differences were observed in the reversion of colon length reductions or in the degree of reductions in DAI scores, the restoration of body weight by ED amino acids was significantly enhanced compared to that with EN enriched in short peptides and intact proteins. The mice's body weight improvement started on day 28 for ED amino acids treatment group, but for the other two treatment groups, the mice's body weight did not improve until day 38. Therefore, with the most effective formula, we then investigated the potential anti-inflammatory mechanism of ED amino acids underlying the therapeutic effects on inducible colitis.

### 3.2. An Elemental Diet Enriched in Amino Acids Suppresses Proinflammatory Cytokines in the Colon

**Figure 2:**
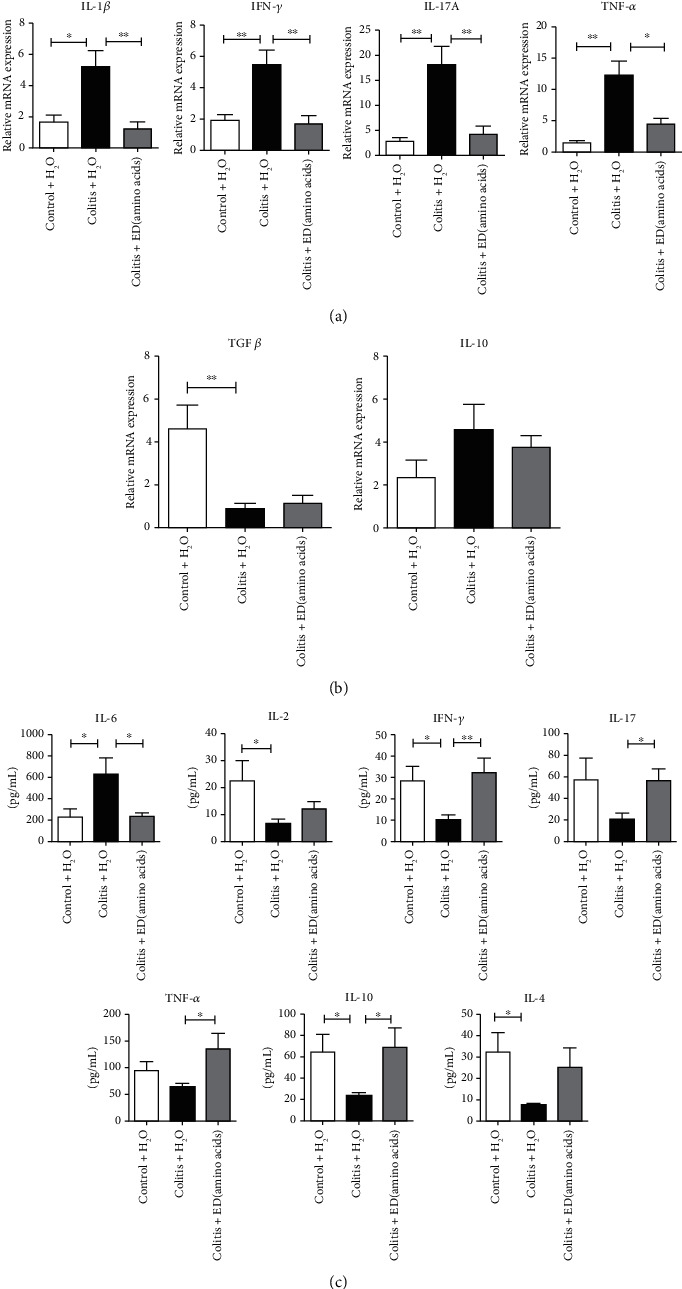
An elemental diet enriched in amino acids suppresses proinflammatory cytokines in the colon. The mRNA levels of proinflammatory (a) and anti-inflammatory cytokines (b) relative to the control GAPDH (*n* = 5 per group) in individual groups. (c) Levels of multiple cytokines produced in the serum samples of individual groups were analyzed by flow cytometry using a CBA mouse Th1/Th2/Th17 cytokine kit. Data are expressed as the mean ± SEM of each group from three separate experiments. ∗*p* < 0.05, ∗∗*p* < 0.01.

First, we found that the colonic mRNA expression levels of the proinflammatory mediators *IL-1β*, *IFN-γ*, *IL-17A*, and *TNF-α*, which had been confirmed to be involved in IBD, were upregulated notably in colitis mice compared to levels in normal controls (Figure 2(a)). Meanwhile, the expression levels of *TGFβ* mRNA, encoding an important cytokine that suppresses inflammation, were reduced dramatically in colitis mice compared to those in the control group (Figure 2(b)). Furthermore, treatment with ED amino acids was only found to downregulate these upregulated genes, namely *IL-1β*, *IFN-γ*, *IL-17A*, and *TNF-α* but had no impact on *TGFβ* and *IL-10* mRNA transcripts in the intestinal tissues (Figures 2(a) and 2(b)). In conclusion, ED amino acids resulted in predominant resistance to the release of inflammatory cytokines in the colon.

Additionally, to determine the influence of ED amino acid treatment on inflammation in the peripheral circulation, the protein levels of several cytokines were measured in the serum from each group using a CBA Mouse Th1/Th2/Th17 Cytokine Kit. DSS treatment elevated the levels of IL-6 and reduced IL-2 and IFN-*γ* levels but had no effect on IL-17A and TNF-*α* (Figure 2(c)). DSS treatment also decreased the basal level of the anti-inflammatory meditators IL-10 and IL-4 (Figure 2(c)). However, the treatment of colitis mice with ED amino acids effectively eliminated the DSS-mediated increase in IL-6 and reduction in IL-10 protein levels in the serum. ED amino acids also resulted in an increase in IFN-*γ*, TNF-*α*, and IL-17A levels but had no effect on IL-2 and IL-4 levels in colitis mice. Overall, ED amino acids exerted a bidirectional regulatory effect on proinflammatory and anti-inflammatory cytokine release in the peripheral circulation.

### 3.3. An Elemental Diet Enriched in Amino Acids Affects the Accumulation of Intestinal Lamina Propria Dendritic Cells in Chronic Colitis Mice

It has been reported that IFN regulatory factor 4- (IRF4-) dependent CD103^+^CD11b^+^ dendritic cells (DCs) have a role in the generation of intestinal T helper 17 (Th17) cells, and evidence was provided that CD103^+^CD11b^+^ DCs are important for Th17 cell differentiation in the intestinal draining MLN [15]. In addition, natural killer cells (NK) are the main component of innate immunity, but whether ED amino acids have a regulatory role in NK cells and DC cells in a chronic colitis model is not clearly understood. We next evaluated the effects of ED amino acids on the CD103^+^CD11b^+^ DC and CD3^−^ NK1.1^+^ NK cells by flow cytometry.

**Figure 3:**
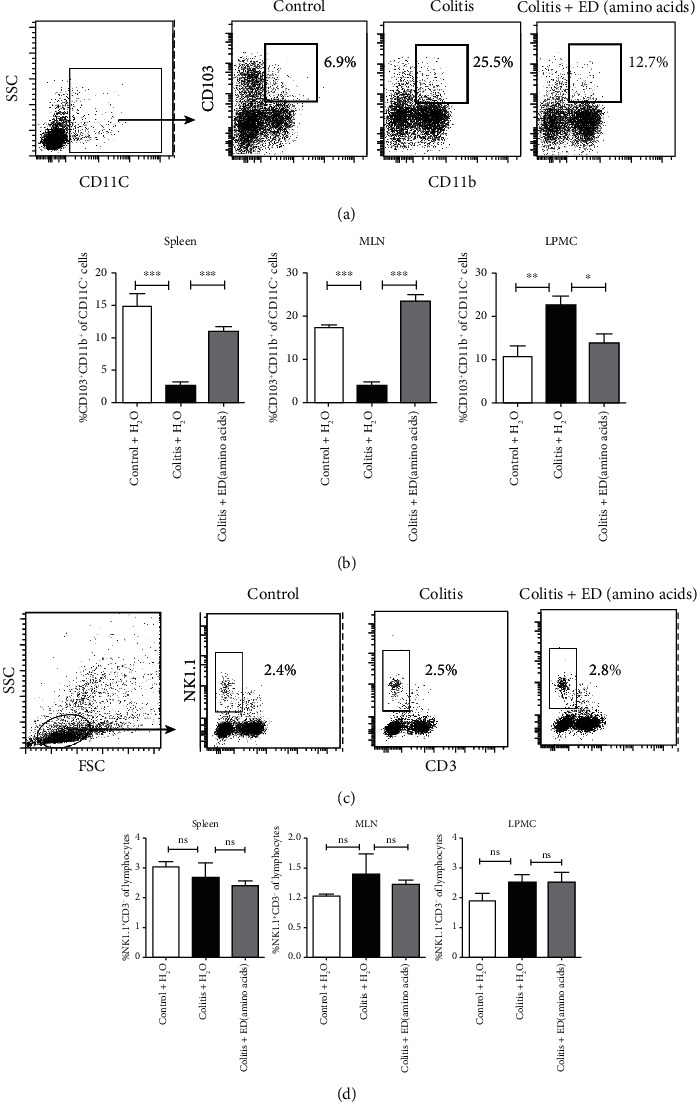
An elemental diet enriched in amino acids affects the accumulation of intestinal lamina propria dendritic cells (DCs) in chronic colitis mice. Mouse splenic mononuclear cells (MNCs), mesenteric lymph nodes (MLNs), and colonic lamina propria mononuclear cells (LPMCs) were isolated from individual groups of mice. Representative flow cytometric analyses of CD11C^+^CD103^+^CD11b^+^ DCs (a); MNCs were gated in CD11C^+^, and the percentages of CD103^+^CD11b^+^ DCs were analyzed in the spleen; MLNs and LPMCs in different groups of mice (b). Representative flow cytometric analyses of CD3^−^ NK1.1^+^ NK cells (c) and NK proportions in MNCs (d) in the spleen, MLN, and LPMCs of different groups of mice (*n* = 5 per group). Data are representative images or expressed as the mean ± SEM of each group from three separate experiments. ∗*p* < 0.05, ∗∗*p* < 0.01, ∗∗∗*p* < 0.001; ns: not significant.

We found that the frequency of CD11C^+^CD103^+^CD11b^+^ DC cells was significantly increased in LPMCs but was dramatically reduced in the spleen and MLN in colitis mice when compared to those in normal controls (Figures 3(a) and 3(b)). As one of our major findings, ED amino acid administration led to a significant decrease in the migration of CD103^+^CD11b^+^ DCs into the colon lamina propria (LP) of colitis mice (Figure 3(b)). Moreover, ED amino acid administration was able to affect the migration of LP CD103^+^CD11b^+^ DCs, which are responsible for Th17 cell differentiation in the intestine, further indicating that ED amino acids might have an effect on the Th17 response during intestinal mucosal immunity. However, no statistical differences in NK cells among different organs of control, colitis, and treated groups were found (Figures 3(c) and 3(d)). This suggested that NK cells might not be the predominant cell that contributes to the pathological destruction of the colon in DSS-induced chronic colitis.

### 3.4. An Elemental Diet Enriched in Amino Acids Inhibits the Predominant Th1/Th17 Responses in the Colon of Colitis-Bearing Mice

**Figure 4:**
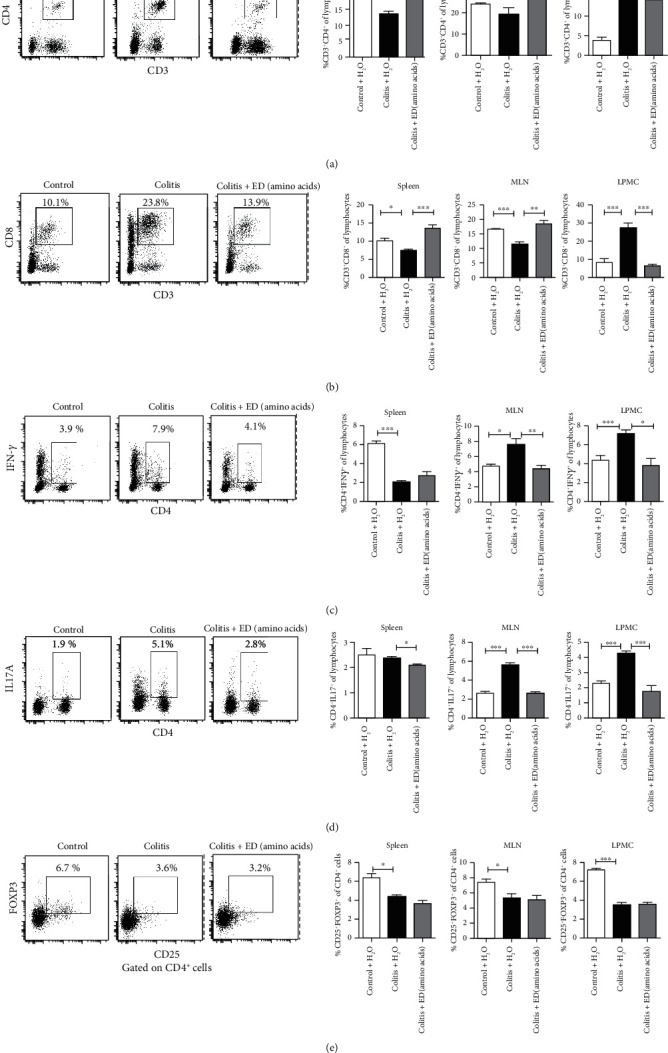
An elemental diet enriched in amino acids inhibits the predominant Th1/Th17 responses in the colon of colitis-bearing mice. Flow cytometric analysis of CD3^+^CD4^+^ T (a), CD3^+^CD8^+^ T (b), CD4^+^ IFN-*γ*^+^ Th1 (c), and CD4^+^ IL17A^+^ Th17 cells (d) in the spleen, mesenteric lymph node (MLNs), and lamina propria mononuclear cells (LPMCs) from individual groups of mice were calculated. CD4^+^CD25^+^FoxP3^+^ Treg (e) frequency in CD4^+^ T cells was examined. Representative flow cytometric dot plots (left) and percentages of positive cells (right) are depicted (*n* = 5 per group). Data are representative images or expressed as the mean ± SEM of each group from three separate experiments. ∗*p* < 0.05, ∗∗*p* < 0.01, ∗∗∗*p* < 0.001.

To further assess the effects of ED amino acids on the Th1/Th17/regulatory T (Treg) cell profile, we characterized the frequency of different subsets of T cells by flow cytometry. In comparison to that in the control mice, a significantly lower frequency of splenic and MLN CD4^+^ and CD8^+^ cells and Tregs, as well as splenic Th1 cells, was detected in the colitis group (Figures 4(a), 4(b), 4(c), and 4(e)). Further, a significantly increased frequency of CD4^+^, CD8^+^ T, CD4^+^ IFN-*γ*^+^ Th1, and CD4^+^ IL17^+^ Th17 cells, but decreased Tregs, were detected in the colon mucosal LP of the colitis group (Figures 4(a)–4(e)), indicating that CD4^+^, CD8^+^, Th1, and Th17 cells infiltrate into the LP of the colon during the process of chronic colitis. As we found, DSS application affected the cellular composition of the colon environment. Interestingly, treatment with ED amino acids significantly eliminated the colitis-mediated increase in the frequency of CD4^+^, CD8^+^, Th1, and Th17 cells in the colon mucosal LP of mice (Figures 4(a)–4(d)). However, ED amino acid application was unable to restore the frequency of Tregs in the colitis mice (Figure 4(e)), and these cells play a key role in the maintenance of immune homeostasis to prevent IBD [16, 17]. Our novel findings indicated that ED amino acids have a potential regulatory effect on Th1 and Th17 responses in this model.

### 3.5. An Elemental Diet Enriched in Amino Acids Prevents Colonic Epithelial Barrier Dysfunction Induced by DSS, Maintaining the Expression of Tight Junction Proteins

**Figure 5:**
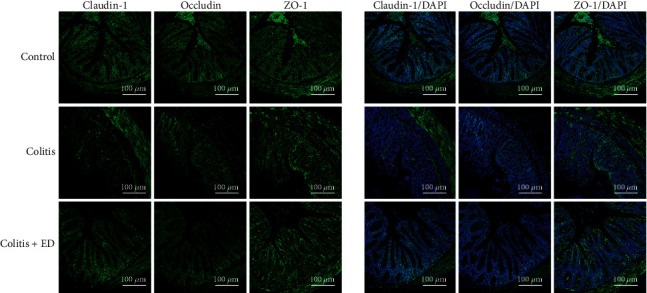
An elemental diet enriched in amino acids prevents colonic epithelial barrier dysfunction induced by dextran sulfate sodium (DSS), maintaining the expression of tight junction proteins. Immunofluorescence staining of claudin-1, occludin, and ZO-1 in the intestinal tissues from different groups of mice with positive expression (green) determined by confocal microscopy. Cell nuclei were stained with DAPI (blue). Scale bar, 100 *μ*m.

We next investigated whether ED amino acids have an effect on intestinal permeability by detecting the expression levels of three major tight junction proteins. Immunofluorescence analysis of claudin-1, occludin, and ZO-1 showed ample staining in the membrane, which was found mainly between epithelial cells of normal control mouse colons (Figure 5). We found that occludin, ZO-1, and claudin-1 expression levels in the colon were significantly reduced in colitis mice, and especially claudin-1. Interestingly, treatment with ED amino acids markedly decreased epithelial architectural derangements and restored the expression of occludin, ZO-1, and claudin-1 protein levels in colitis mice (Figure 5). Together, ED amino acids have a protective effect, repairing the disrupted barrier function.

## 4. Discussion

Recently, several probable mechanisms underlying the use of EN for IBD therapy were proposed. It was suggested that EN could decrease hypermetabolism in active CD patients [18]. Further, EN was found to ameliorate mesenteric fat alterations in adult CD by restoring adipocyte morphology and diminishing the inflammatory environment of mesenteric fat [19]. Moreover, EN changes the intestinal microbiota in IL-10^−/−^ cell-transferred colitis mice, and especially the lactic acid bacteria composition [20]. Modification of the microbiota by EN might be responsible for the inhibition of colitis. However, whether it manages the disordered mucosal immune response during the process of colitis is not clearly understood.

In the present study, we employed a mouse model of DSS-induced chronic colitis to investigate the therapeutic effect of EN on disease severity, the cellular composition of the colon environment, and epithelial barrier functions. First, we demonstrated that three EN formulas effectively attenuated colitis severity and mucosal lesions in experimental chronic colitis mice, consistent with previous reports [10, 21]. Interestingly, we found that ED amino acids restored the body weight reductions better than the other two EN formulas. Among the three EN formulas, amino acid-based ED has the lowest fat content (0.17 g/100 kcal vs. 3.53 g/100 kcal vs. 1.70 g/100 kcal, Table 1). Fat can cause intestinal peristalsis; therefore, it is believed that low-fat diets are beneficial for IBD patients, based on their ability to keep the intestinal tract still. It was proposed that the fat content and fat composition of EN are responsible for their therapeutic effect in active CD patients [22]. Moreover, Bamba et al. reported that active CD patients receiving the low-fat diet are associated with the highest remission rate. In contrast, when the fat content consisted of a high amount of long-chain triglycerides, the therapeutic effect against active CD decreased [23]. In the case of exclusive enteral nutrition, only ED amino acid formulas can provide a low-fat diet at lower than 20 g/day. Therefore, it is reasonable to assume that the outstanding anti-inflammatory effect of ED amino acids might be due to the low fat content.

Although a previous study by Souza et al. revealed that the consumption of amino acid diets can exacerbate experimental colitis [24], clinical outcomes suggest the opposite. Exclusive enteral nutrition with ED amino acids has been used extensively for the application of IBD therapy, and this has been proven effective in achieving and maintaining remission [25, 26]. During an inflammatory flare, IBD patients are at a high risk of nutrient depletion, and this is particularly true for children [27]. An improvement in malnutrition in IBD patients was observed after ED amino acid treatment [28]. In addition, the amino acid composition of ED is considered hypoallergenic [12], and some amino acids such as histidine, tryptophan, methionine, glutamine, cysteine, and arginine have beneficial effects [29]. Dietary interventions with specific amino acids in IBD can reduce inflammation, oxidative stress, and apoptosis in the gut, which is responsible for their anti-inflammatory activity, as reviewed by Zhang et al. [30]. Moreover, Faure et al. reported that intestinal tissue repair processes increase the host's need for specific nutrients, and diets supplemented with specific amino acids containing L-threonine, L-serine, L-proline, and L-cysteine strongly stimulate mucin synthesis in DSS-treated rats, suggesting the replenishment of mucins in the damaged mucosa [31]. Thus, increasing specific amino acid supplementation might affect epithelial protection and repair.

Next, the regulatory effects of ED amino acids on immune cell profiles in the local microenvironment were investigated. Although the pathogenic process of IBD is unclear, an imbalance in proinflammatory and regulatory CD4^+^ T cell responses and damage to the epithelial barrier, which is the first-line defense of the mucosal immune system, are crucial for the development and progression of IBD [16, 32–34]. As a result, we subsequently analyzed the cellular composition of the colon environment (including CD103^+^CD11b^+^ DC, CD3^−^ NK1.1^+^ NK, CD4^+^, and CD8^+^ T cells) and found the significant infiltration of CD4^+^ and CD8^+^ T cells and the migration of DC cells into the colon LP of colitis mice. Interestingly, ED amino acids can reduce DC and CD4^+^ and CD8^+^ T cell accumulation, which is accompanied by the resolution of inflammation. More importantly, predominant Th1 and Th17 responses present as colitis-related abundant CD4^+^ IFN-*γ*^+^ Th1 and CD4^+^ IL17^+^ Th17 cells that infiltrate into the colon mucosa LP, and colitis-mediated upregulated *IFN-γ* and *IL-17A* mRNA transcripts in the intestinal tissues were found in colitis mice. Encouraging results also indicated that ED amino acids effectively inhibit the predominant Th1/Th17 responses by downregulating Th1 and Th17 cells and colonic *IFN-γ* and *IL-17A* mRNA transcripts. Such results provided supporting data to explain how EN regulates cytokines and the immune response disorders, which might reflect one aspect of the mucosal immune microenvironment in IBD.

In the current study, ED amino acids were deemed more effective with respect to their anti-inflammatory actions, rather than the restoration of immune homeostasis. ED amino acids reduced LPMC CD4^+^, CD8^+^ T cells, DC cells, and Th1 and Th17 cells and reduced the mRNAs transcripts of colonic upregulated proinflammatory cytokines in colitis mice but had no effect on the reduction of Treg frequency and the anti-inflammatory, colonic cytokine mRNA transcripts. ED amino acids reduced the colitis-mediated upregulation of LPMC CD103^+^CD11b^+^ DCs, which are responsible for Th17 cell differentiation in the intestine [15], further indicating that ED amino acids might have an effect on innate and adaptive immune responses in the colon. However, it remains unclear whether ED amino acids only contributed to the reduction of CD103^+^CD11b^+^ DC cell-derived, mucosal Th17 cell differentiation or whether ED amino acids were simultaneously directly responsible for suppressing Th17 cell quantity. Because of the complex composition of EN, in vitro studies to clarify and differentiate its effect on immune cells are difficult to implement. Overall, we provide evidence to further support the outstanding anti-inflammatory effects of ED amino acids, which are not only due to the low fat content or amino acid composition, for the application to IBD.

Patients with IBD display increased intestinal epithelial permeability and disrupted barrier function [35]. In addition, correcting the epithelial barrier defect in IL-10^−/−^ mice is beneficial to attenuate colitis [36]. Cytokines including IL-6, IL-1*β*, IFN-*γ*, and TNF-*α* promote disruption of the intestinal tight junctions [37], resulting in increased mucosal permeability and a vicious cycle of reactivation of the local inflammatory response [38, 39]. The intestinal epithelial barrier function relies on selective permeability, which depends on tight junctions [40]. Interestingly, we found that colitis results in the upregulation of *IL-1β*, *IFN-γ*, and *TNF-α* mRNA transcripts and the reduced expression of tight junction proteins. In this context, ED amino acids can prevent the reduction in tight junction protein expression; this effect might be partly due to the predominant inflammation-inhibiting action that occurs by reducing the generation of inflammatory mediators.

## 5. Conclusions

In conclusion, our data indicated that treatment with ED amino acids significantly mitigates DSS-induced colitis and improves mucosal lesions, maintaining the expression of tight junction proteins, suppressing the release of colonic proinflammatory mediators, and resulting in the bidirectional regulation of proinflammatory and anti-inflammatory cytokine release in the peripheral circulation and the suppression of Th1/Th17 responses. Our findings provide new evidence to support the outstanding anti-inflammatory effects of ED amino acids in a mouse model of colitis.

## Figures and Tables

**Table 1:** A comparison of three different EN formulas.

Name	Nitrogen source	Fat (g/100 kcal)	Protein (g/100 kcal)	Carbohydrate (g/100 kcal)
Enteral nutritional powder (AA)	17 amino acids	0.17	4.7	21.14
Enteral nutritional suspension (SP)	Whey protein hydrolysates	1.7	4.0	17.6
Enteral nutritional powder (TP)	Casein-calcium, casein-sodium, soybean protein	3.53	3.53	13.5

## Data Availability

The data used to support the findings of this study are available from the corresponding author upon request.

## Supplementary Materials

Supplementary Materials Table S1: the composition of three different EN formulas.
