# Supplementary material for: Elemental Diet Enriched with Amino Acids Alleviates Mucosal Inflammatory Response and Prevents Colonic Epithelial Barrier Dysfunction in Mice with DSS-Induced Chronic Colitis
Source: J Immunol Res. 2020 Aug 14;2020:9430763. doi: 10.1155/2020/9430763 (PMC7443247; doi:10.1155/2020/9430763)
Supplement: Supplementary Materials — Table S1: the composition of three different EN formulas. [file 9430763.f1.docx]

| Generic name | Enteral Nutritional Powder (AA) | Enteral Nutritional Powder (TP) | Enteral Nutritional Suspension (SP) |
| --- | --- | --- | --- |
| Composition (Calculated bv 1 packing unit) | | | |
| Fat (g) | 0.51 | 98.4 | 8.5 |
| Carbonhydrates (g) | 63.41 | 242.8 | 88 |
| Nitrogen source | 17 Amino acids (L-Isoleucine, 0.642 g; L-Leucine, 0.899 g; Lysine hydrochloride, 0.888 g; L-Methionine, 0.648 g; L-Phenylalanine, 0.871 g; L-Threonine, 0.523 g; L-Tryptophan, 0.151 g; L-Valine, 0.701 g; L-Histidine hydrochloride monohydrate, 0.501 g; L-Arginine hydrochloride, 1.125 g; L-Alanine, 0.899 g; L-Aspartic acid magnesium potassium, 1.036 g; L-Aspartic acid sodium monohydrate, 0.867 g; L-Glutamine, 1.932 g; Aminoacetic acid, 0.505 g; L-Proline, 0.63 g; L-Serine, 1.159 g; L-Tyrosine, 0.11) | Casein-calcium，Casein-sodium，Soybean protein (63.6 g) | Whey protein hydrolysates (20g) |
| Vitmins | | | |
| Vit A (IU) | 648(VitA acetate) | 4680 | 1365.3 |
| Vit B1 (mg) | 0.194 | 2.88 | 0.75 |
| Vit B2 (mg) | 0.256(Riboflavin sodium phosphate) | 3.2 | 0.8 |
| Vit B6 (mg) | 0.267 | 4 | 0.85 |
| Vit B12 (μg) | 0.7 | 12.4 | 1.05 |
| Vit C (mg) | 7.8 | 272 | 50 |
| Vit D (μg) | 1.3 (Vit D2) | 9.5 (Vit D3) | 3.5 |
| Vit E (mg) | 3.3 | 42.8 | 9.7 |
| Vit K (μg) | 9 (Vit K1) | 72 | 26.5 |
| Nicotinic acid (mg) | 2.2 (nicotinamide) | 40 | 4 |
| Folic acid (μg) | 44 | 800 | 135 |
| Pantothenic acid (mg) | 1.19 (Calcium Pantothenate) | 20 | 2.65 |
| Biotin (μg) | 39 | 600 | 20 |
| Choline (mg) | 17.93 (Choline Bitartrate) | 544 | 185 |
| Minerals | | | |
| Fe (mg) | 15.5(Ferrous gluconate ) | 17.48 | 8 |
| Ca (mg) | 825(Calcium Glycerophosphate ) | 920 | 400 |
| P (mg) | 825(Calcium Glycerophosphate ) 0.256(Riboflavin sodium phosphate) | 920 | 360 |
| Na (mg) | 616(Sodium citrate) 867(L-Aspartic acid sodium monohydrate) 0.256(Riboflavin sodium phosphate | 1440 | 500 |
| K (mg) | 1036(L-Aspartic acid magnesium potassium ) 150(potassium chloride) 19.6(Potassium iodide) | 2680 | 750 |
| Cl (mg) | 150(potassium chloride) | 2440 | 625 |
| Mg (mg) | 1036(L-Aspartic acid magnesium potassium ) 1.3(Manganese sulfate) | 360 | 115 |
| I (μg) | 19.6(Potassium iodide) | 136 | 65 |
| Cu (μg) | 820(Copper sulfate) | 2080 | 900 |
| Mn (μg) | 1300(Manganese sulfate with 5 water molecules) | 4800 | 1650 |
| Zn (mg) | 7.88(Zinc sulfate) | 21.6 | 6 |
| Se (μg) | —— | 80 | 28.5 |
| Cr (μg) | —— | 80 | 33.5 |
| Mo (μg) | —— | 152 | 50 |
| Fluorine C | —— | —— | 500 |
| Taurine (mg) | —— | —— | 50 |
| Average parameters (g/100kcal) | | | |
| Protein/Amino acid | 4.70 | 3.53 | 4.00 |
| Fat | 0.17 | 3.53 | 1.70 |
| Carbohydrate | 21.14 | 13.50 | 17.60 |

**Supplementary Table 1.** The composition of three different EN formulas
